# Non-Viral Related Liver Enzymes Elevation After Kidney Transplantation

**DOI:** 10.5812/hepatmon.9036

**Published:** 2014-02-05

**Authors:** Behzad Einollahi, Alireza Ghadian, Ebrahim Ghamar-Chehreh, Seyed Moayed Alavian

**Affiliations:** 1 Nephrology and Urology Research Center, Baqiyatallah University of Medical Sciences, Tehran, IR Iran; 2 Baqiyatallah Research Center for Gastroenterology and Liver Diseases, Baqiyatallah University of Medical Sciences, Tehran, IR Iran; 3 Middle East Liver Diseases Center (MELD), Tehran, IR Iran

**Keywords:** Kidney, Transplantation, Cyclosporine, Aspartate aminotransferase, Alanine aminotransferase

## Abstract

**Background:**

Liver enzymes elevations (LEE) can be observed after kidney transplantation due to multifactorial causes.

**Objectives:**

We performed a retrospective study on 1589 kidney transplants, 971 male and 618 female, who were hepatitis B surface antigen (HBsAg) and hepatitis C virus-antibody (HCV Ab) negative, and had no other liver diseases, to detect the prevalence of LEE and its risk factors in these patients between May 2008 and May 2010.

**Patients and methods:**

Liver enzymes and other biochemical parameters were measured in all recipients. Patients were divided into three groups, according to laboratory test time since transplantation: Group I, less than 3 months, Group II, 4 - 12 months after transplantation, and Group III, more than one year post-transplantation.

**Results:**

The highest LEE was more frequent in older patients (P < 0.001) and male individuals (P < 0.001). Aspartate aminotransferase (AST) and alanine aminotransferase (ALT) levels were higher in patients who received kidneys from deceased donors (10.4% and 23.8%, respectively) as compared to living donor transplants (5.6% and 14.8%, respectively) (P < 0.001). The elevation of ALT was the liver enzyme abnormality after kidney transplantation with the highest prevalence (34.3%). The levels of ALT and AST were significantly elevated within the first 3 months after transplantation, followed by the 4-12 months period (P < 0.001). There was a reverse correlation between liver enzyme levels and renal allograft function in both univariate and linear regression analyses. This correlation increased over time. There was also a significant relation between cyclosporine blood levels and liver enzyme values in the univariate analysis. However, this relationship was attenuated over time. Elevated liver enzymes also correlated with anemia.

**Conclusions:**

The LEE is a common finding among kidney transplant recipients. Serial monitoring of aminotransferases, particularly ALT, should be performed in all patients after kidney transplantation.

## 1. Background

Chronic liver disease, usually caused by hepatitis B virus (HBV) and hepatitis C virus (HCV), is an important threatening complication after kidney transplantation (1, 2). The HCV infection is the most frequent reason of post-transplant hepatitis (3). Other viral infections, such as herpes simplex and cytomegalovirus (CMV), and immunosuppressive agents used in the kidney transplant patients, should be considered in the differential diagnosis of post-transplant hepatic dysfunction. The decrease of prevalence and incidence of HBV and HCV infections in hemodialysis and renal transplanted patients during recent years sensitize the physicians in nephrology departments to investigate for other causes of liver diseases in their patients (1, 4-7).

Liver enzymes elevations (LEE), including aspartate aminotransferase (AST), alanine aminotransferase (ALT), and alkaline phosphatase (ALP), are indicators of hepatocellular injury (8). Patients with increased levels of serum aminotransferases and/or gamma-glutamyl transpeptidase, should be questioned about ingestion of alcohol and hepatotoxic drugs (9). Thus, serum aminotransferase levels should be monitored in kidney transplant recipients who used potential hepatotoxic agents such as immunosuppressive drugs, statins, allopurinol, etc. There are, however, surprisingly few studies to determine the prevalence of LEE after kidney transplantation.

## 2. Objectives

This retrospective study was performed a on a reasonable number of kidney transplants to detect the prevalence of LEE and its risk factors in these patients, between May 2008 and May 2010.

## 3. Patients and Methods

### 3.1. Study Population

We enrolled 1589 renal transplant recipients (RTRs) who were negative for hepatitis B surface antigen (HBsAg) and hepatitis C virus-antibody (HCV Ab), and had no other liver diseases. Blood samples were taken from all cases (7632 samples) for the measurements of routine parameters such as liver enzymes, serum creatinine (SCr), cyclosporine (CsA) through level (C0) and 2-hour post-dose level of CsA (C2).

Patients were divided into three groups, according to laboratory test time since transplantation: Group I, less than 3 months, Group II, 4-12 months after transplantation, and Group III, more than 1 year post-transplantation.

### 3.2. Data Collection

Demographic variables and other biochemical measurements were gender of recipient and donor, age of recipient and donor, donor sources (living and deceased), blood urea, lipid profiles, fasting blood sugar (FBS), uric acid and hemoglobin (Hb). All recipients were followed for at least 6 months. The study was approved by the local research center ethics committee.

### 3.3. Protocol of Treatment and Follow Up

Immunosuppression maintenance in all recipients was primarily based on cyclosporine (CsA). The majority of patients also received mycophenolate mofetil/azathioprine and prednisolone. Induction therapy with anti-thymocyte globulin was routinely administered in highly sensitized cases, such as patients with high panel reactive antibody and second or third transplantation. 

All patients were clinically examined and a blood sample was taken for routine laboratory test at a weekly interval until 1 month, then at two weeks period up to 3 months, followed by monthly test for 1 year and every 1-2 months subsequently.

### 3.4. Definitions

We considered the upper limit of normal for aminotransferases (ALT and AST) at 40 IU/L and ALP to 316 IU/L. Patients with renal allograft dysfunction (SCr > 2 mg/dl) were considered to have high ALT levels if the ALT level was > 27 IU/l. It has been shown that 27-IU/l is the upper limit of ALT for dialysis patients without liver disease (10).

### 3.5. Statistical Analysis

Data were analyzed using SPSS for Windows version 17.0 (SPSS Inc., Chicago, Illinois, The USA) and the quantitative results were presented as mean ± SD, while qualitative variables were expressed by number and percentage. The Kolmogorov-Smirnov test (K-S test) showed that AST and ALT levels were not distributed normally. Consequently, Spearman's correlation analysis was used to assess the correlations between liver enzymes levels with numeric variables. Chi square test was used to compare qualitative data. Comparisons of variables, such as AST, ALT and ALP, between male and female recipients, were performed using the Mann-Whitney U-test. Multiple logistic regression was also performed for abnormal ALT, considered as a categorical factor. A result was considered statistically significant when P < 0.05.

## 4. Results

### 4.1. Demographic Setting

A total of 1589 RTRs, 971 males and 618 females, were included in this study. Table 1 shows the demographic data of patients. The majority of patients received a kidney from a living unrelated donor (Table 1). The older patients had higher liver enzymes level, as compared to younger individuals (Table 2). Liver enzymes value was higher in male compared with female individuals (Table 3). The levels of AST and ALT were higher in patients who received the kidney from deceased donors, as compared to living donor transplants (10.4% and 23.8%, in deceased renal transplantation, respectively, versus 5.6% and 14.8%, in living renal transplantation, respectively, P < 0.001). Conversely, ALP level was greater in living renal transplants than in those who received the kidney from a deceased donor (17% versus 13%, P = 0.006).

**Table 1.** Demographic Data of Transplant Patients

Variable	Amounts
**Recipients Sex, Male/Female, %**	61/39
**Donors Sex, Male/Female, %**	83/17
**Donor Sources, LRD ^a^/LURD ^a^/Deceased, %**	8.2/84.9/6.9
**Times of Tx ^a^, 1/2/3**	1529/53/7
**AST ^a^, IU/L, Mean ± SD**	22.2 ± 29.0
**ALT ^a^, IU/L, Mean ± SD**	30.4 ± 46.3
**TB, Mean ± SD**	1.1 ± 3.2
**ALP ^a^, IU/L, Mean ± SD**	243.3 ± 188.9
**Trough Level of CsA ^a^, ng/mL, Mean ± SD**	155.1 ± 98.1
**2-h Dose CsA, ng/ml, Mean ± SD**	518.7 ± 174.3
**Age of Recipients, Mean ± SD, y**	38 ± 15
**Age of Donors, Mean ± SD, y**	28 ± 6
**BUN ^a^, mg/dL, Mean ± SD**	52 ± 31
**Serum Cr ^a^, mg/dL, Mean ± SD**	1.6 ± 0.9
**FBS ^a^, mg/dL, Mean ± SD**	103 ± 46
**HDL ^a^ Cholesterol, mg/dL, Mean ± SD**	48.2 ± 15.7
**LDL ^a^ Cholesterol, mg/dL, Mean ± SD**	102.2 ± 34.4
**Uric Acid, mg/dL, Mean ± SD**	6.1 ± 1.8
**Hb ^a^, g/dl, Mean ± SD**	12.6 ± 2.2

^a^ Abbreviations: ALP, alkaline phosphatase; ALT, alanine aminotransferase; AST, aspartate transaminase; BUN, blood urea nitrogen; Cr, creatinine; CsA, cyclosporine; FBS, fasting blood sugar; Hb, hemoglobin; HDL, high density lipoprotein; LDL, low density lipoprotein; LRD, living related donor; LURD, living unrelated donor; TB, total bilirubin; Tx, transplantation.

**Table 2.** Correlation Between Liver Enzyme Levels and Other Parameters

Variables	P value (Correlation Coefficient)			
	AST ^a^	ALT ^a^	TB ^a^	ALP ^a^
**Trough Level of CsA ^a^**	< 0.001 ^b^ (0.17)	< 0.001 ^b^ (0.29)	< 0.001 ^b^ (0.11)	0.1 (-0.02)
**2-hours post dose CsA**	< 0.001 ^b^ (0.13)	< 0.001 ^b^ (0.23)	< 0.001 ^b^ (0.14)	0.001 ^b^ (-0.07)
**Age of Recipient**	0.06 (0.02)	< 0.001 ^b^ (0.07)	< 0.001 ^b^ (0.09)	< 0.001 ^b^ (-0.06)
**Age of Donor**	0.6 (-0.007)	0.8 (-0.004)	0.08 (0.04)	0.1 (0.02)
**Serum Creatinine**	< 0.001 ^b^ (-0.12)	< 0.001 ^b^ (-0.07)	< 0.001 ^b^ (-0.02)	< 0.001 ^b^ (-0.07)
**FBS ** ^**a**^	0.6 (0.006)	< 0.001^ b^ (0.09)	0.083 (0.03)	< 0.001 ^b^ (0.1)
**HDL ^a^ Cholesterol**	0.15 (0.02)	0.08 (0.03)	< 0.001 ^b^ (0.1)	< 0.001 ^b^ (-0.09)
**LDL ^a^Cholesterol**	0.4 (0.01)	0.06 (0.03)	0.1 (-0.03)	0.008 ^b^ (0.04)
**Uric Acid**	0.004 ^b^ (-0.04)	< 0.001 ^b^ (-0.09)	0.6 (0.009)	0.09 (-0.02)
**Hb ** ^**a**^	< 0.001 ^b^ (0.09)	0.001 ^b^ (0.06)	< 0.001 ^b^ (0.4)	< 0.001 ^b^ (0.09)

^a^ Abbreviations: ALP, alkaline phosphatase; ALT, alanine aminotransferase; AST, aspartate transaminase ; CsA, cyclosporine; Hb, hemoglobin; HDL, high density lipoprotein; LDL, low density lipoprotein; TB, total bilirubin.

^b^ P value < 0.05.

**Table 3.** Comparisons of Liver Enzyme Levels in Both Genders

Liver enzymes	mean ± SD	P value
**AST ^a^**		< 0.001 ^b^
Male	22.9 ± 33.9	
Female	21.0 ± 17.8	
**ALT ** ^**a**^		< 0.001 ^b^
Male	32.2 ± 50.8	
Female	27.5 ± 37.6	
**TB ^a^**		< 0.001 ^b^
Male	1.2 ± 2.6	
Female	0.9 ± 4.1	
**ALP ** ^**a**^		< 0.001 ^b^
Male	247.1 ± 178.1	
Female	236.6 ± 205.6	

^a^ Abbreviations: ALP, alkaline phosphatase; ALT, alanine aminotransferase; AST, aspartate transaminase; TB, total bilirubin.

^b^ P value < 0.05.

### 4.2. Prevalence

Table 4 demonstrates the prevalence of each liver enzyme abnormality in all patients and in the three groups. The prevalence of ALT elevation was most frequent liver enzyme abnormality after kidney transplantation (Table 4). The levels of ALT and AST were significantly elevated within the first 3 months after transplantation, followed by the 4-12 months period (P < 0.001). The mean of ALT was higher than the mean of AST in our patients (Table 5). However, there was no significant difference in the prevalence of elevated ALP level between the three periods after transplantation (Table 4). The mean value of ALP concentration was higher in the first 3 months after transplantation, when compared with other periods following kidney transplantation (Table 5). 

**Table 4.** Prevalence of Liver Enzymes Elevation After Kidney Transplantation (n = 1589)

Abnormal Variable	Overall	Group I (< 3 months)	Group II (4-12 months)	Group III (> 12 months)	P value
**ALT ^a^, %**	34.3	62.3	53	29	< 0.001^b^
**AST ^a^, %**	6.7	13.4	10.6	5.6	< 0.001 ^b^
**ALP ^a^, %**	16.5	18.9	16.7	16.2	0.2

^a^ Abbreviations: ALP, alkaline phosphatase; ALT, alanine aminotransferase; AST, aspartate transaminase.

^b^ P value < 0.05.

**Table 5.** Mean Value of Liver Enzymes After Kidney Transplantation

Variable	Overall	Group I	Group II	Group III	P value
**ALT ^a^, IU/L, Mean ± SD**	30.4 ± 46.3	52.6 ± 58.0	35.3 ± 27.5	27.1 ± 45.1	< 0.001 ^b^
**AST ** ^**a**^ **, IU/L, Mean ± SD**	22.2 ± 29.0	23.7 ± 10.8	22.5 ± 8.1	17.7 ± 8.4	< 0.001 ^b^
**ALP ** ^**a**^ **, IU/L, Mean ± SD**	243.2 ± 188.9	325.9 ± 295.3	189.2 ± 92.8	214.2 ± 100.3	< 0.001 ^b^

^a^ Abbreviations: ALP, Alkaline phosphatase; ALT, Alanine aminotransferase; AST, Aspartate transaminase.

^b^ P value < 0.05.

### 4.3. Correlation of Liver Enzymes Test and Other Biochemical Parameters

#### 4.3.1. Univariate Analysis

Table 2 shows the correlation of liver enzyme levels with other parameters. There was a reverse correlation between liver enzyme levels and renal allograft function. It means that lower liver enzyme values were seen in patients with renal impairment (1.5 ± 0.9 mg/dL vs. 1.6 ± 0.9 mg/dL for AST, P = 0.009, and 1.5 ± 0.8 mg/dL vs. 1.6 ± 0.9 mg/dL for ALT, P = 0.01). There was a significant relation between CsA blood levels and liver enzyme values. However, ALP level only correlated with C2 blood level (Table 2). 

Elevated liver enzymes were correlated with anemia (Table 2). The concentrations of ALT and ALP were also correlated with FBS (Table 2). Nonetheless, ALP had a weak but significant correlation with the lipid profile (Table 2). 

#### 4.3.2. Multivariate Regression Analysis

In recipients with serum SCr > 2 mg/dL, we considered an ALT level > 27 IU/L as abnormal. Consequently, at multivariate logistic regression, renal impairment was an independent risk factor for abnormal ALT in patients with SCr > 2 mg/dL (Exp(B) = 0.48, 95% CI: 0.32-0.73, P = 0.001).

## 5. Discussion

Elevation of hepatic aminotransferases, ALT and AST, is suggestive of hepatocellular injury. Despite the importance and high prevalence of LEE after kidney transplantation, there are surprisingly few studies on the evaluation of liver enzyme levels among kidney transplant patients and risk factors of their abnormalities after kidney transplantation. In addition, to our knowledge, recent published data in literature on prevalence of liver enzymes abnormalities after kidney transplantation are scarce. The current study shows that, in a quite large sample of kidney transplant patients, liver enzymes are significantly higher in these patients, accounting around 34% for ALT. Liver dysfunction is reported in 7-67% of kidney transplant patients in various studies (11-14). It seems that the cause of this disparity of liver dysfunction frequency is partly due to the diagnostic criteria used, which are different. In patients with SCr < 2 mg/dL, we consider AST and ALT > 40 IU/L as abnormal. However, in recipients with SCr > 2 mg/dL, an ALT > 27 IU/L was considered as abnormal. In our study, ALT was the liver enzyme with the highest prevalence of abnormalities among kidney transplant recipients (34.3%), especially within the first 3 months after transplantation, which is comparable with results from a previous study on 189 kidney transplants, that showed liver enzyme abnormalities in 41% of all patients (8). It is important to note that transient elevation of serum aminotransferases within the first 3 months after transplantation was commonly seen in 22% of all recipients.

Chronic liver disease, which is a frequent complication after renal transplantation, represents the fourth highest cause of death in most series (15). Patients with chronically abnormal liver function studies, with or without hepatitis B surface antigenemia, may experience prolonged allograft function, but also have a high rate of mortality from non-hepatic infection (16, 17). Malekzadeh et al. reported that 9 of 63 (14.2%) children with history of renal transplantation had liver dysfunction (18). Malekzadeh et al. reported that only one of their patients with liver dysfunction had liver function disorder in the first month of the post-transplantation period and other showed liver enzymes abnormality after the third post-transplant month. They noticed that ALT level was higher than AST and only one case had ALP elevation. The etiology of liver dysfunction was considered azathioprine toxicity in eight patients and (CMV) in only one (18). In our study, the majority of patients had hypertransaminasemia in the first 3 months after transplant, unlike the findings of Malekzadeh et al. However, in accordance to their study, we mentioned that ALT serum levels were higher than AST serum levels (Table 3). 

The source of allograft is important in estimating the incidence of liver enzymes elevation. In deceased transplants, this incidence is higher than in living donors (18-20). Similar to these studies, we found that liver enzymes abnormalities were higher and more prevalent in deceased renal transplantations compared to living donors ones.

Lorber et al. in 1987, mentioned that 49% of their renal allograft patients had post-transplant hepatotoxicity with CsA, including hyperbilirubinemia (48% of patients), elevated AST (47%), ALT (73%), LDH (84%), and ALP (59%). Abnormal liver enzymes usually were self-limited in 82% of the cases and generally occurred very soon after transplantation (76%). The serum CsA was relatively high in these patients (225 +/- 17 ng/mL) (21). Their findings are similar, yet much more prevalent than ours. Our patients did not receive azathioprine and we could make abstraction of its effect on liver enzymes. We also observed that elevated liver enzymes are associated with CsA blood levels, renal allograft function and lipid profile. A low grade, transient elevation of serum aminotransferases is commonly seen in patients receiving CsA. If these elevations persist or are severe, a thorough workup, including questioning about viral hepatitis and liver biopsy, is indicated.

### 5.1. Limitations

The main limitations of this study include its retrospective nature, exclusion of non-alcoholic fatty liver disease, as the most common cause of chronic liver disease worldwide, and hepatitis E virus infection, which we did not check in patients with elevated liver enzymes. In addition, liver biopsy and molecular tests were not performed in all kidney transplants with abnormal ALT. It should be noted that liver biopsy is considered as the gold standard of diagnosis of hepatic injury. However, it is invasive, expensive, and may bear potential risks. In addition, the data for the status of opportunistic infections after kidney transplant, ischemia time, ALP bone isoenzyme, medications affecting the liver enzymes such as statins, and body mass index were not available in this study and we could not estimate the impact of these factors on liver enzyme levels after kidney transplantation. The majority of patients also received mycophenolate mofetil and prednisolone. Azathioprine was only used in a small number of patients and we did not investigate the impact of azathioprine on the liver enzymes.

The abnormal liver enzymes are a common finding among kidney transplant patients. The LEE was correlated with age, gender, source of donors, renal allograft function, anemia and CsA blood levels. Serial monitoring of aminotransferases, particularly ALT, should be performed in all patients after kidney transplantation.
